# Supplementary material for: HuBMAPR: an R Client for the HuBMAP Data Portal
Source: bioRxiv. 2024 Sep 27:2024.09.26.615227. Preprint. [Version 1] doi: 10.1101/2024.09.26.615227 (PMC11463621; doi:10.1101/2024.09.26.615227)
Supplement: 1 [file NIHPP2024.09.26.615227v1-supplement-1.pdf]

bioRxiv preprint doi: https://doi.org/10.1101/2024.09.26.615227; this version posted September 27, 2024. The copyright holder for this preprint (which was not certified by peer review) is the author/funder, who has granted bioRxiv a license to display the preprint in perpetuity. It is made available under aCC-BY 4.0 International license.

Supplementary Materials

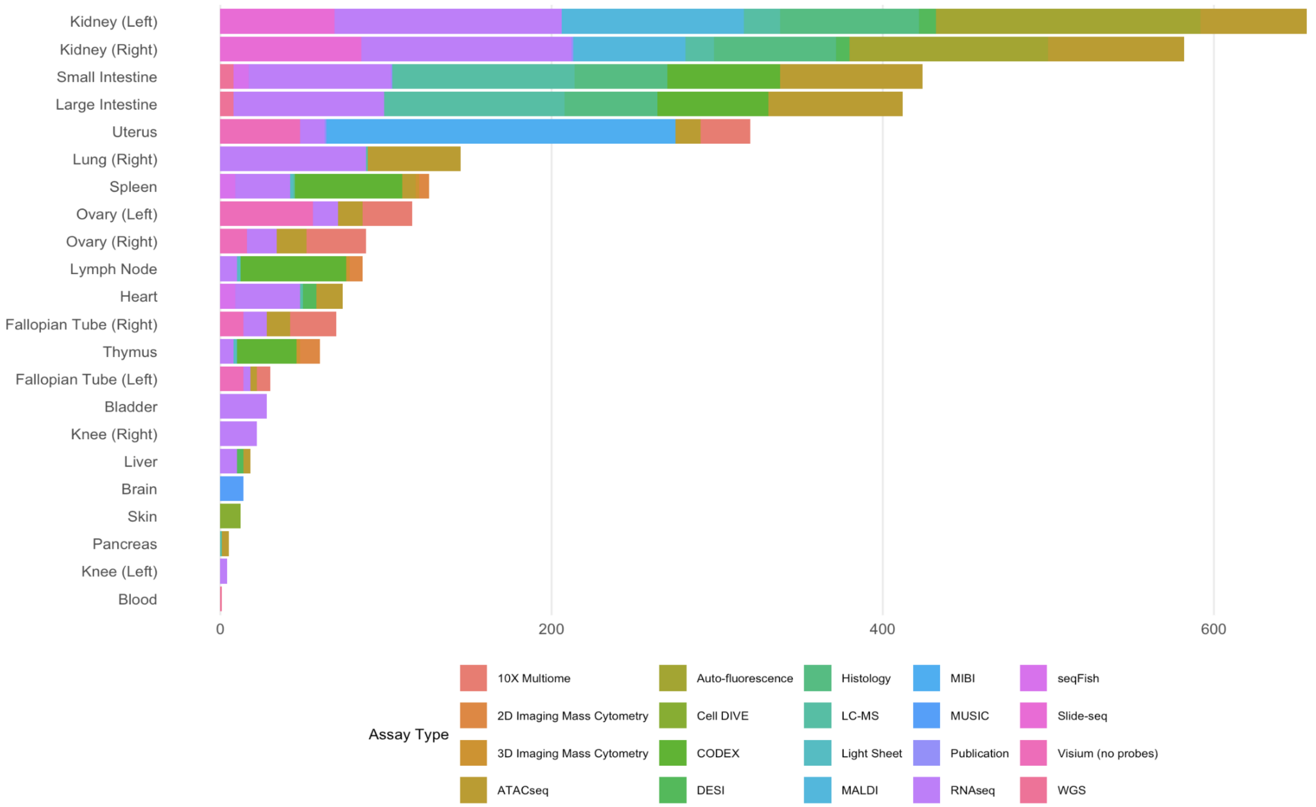

Supplementary Figure S1: **Overview of tissue types in HuBMAP**. HuBMAP Data Portal consists of the original datasets and corresponding support/processed datasets collected from a wide range of organs and processed by multiple assay types (as of August 2024)
